# Comparative Biomechanical Analysis of Two Olecranon Osteotomy Fixation Techniques

**DOI:** 10.1055/s-0045-1811926

**Published:** 2025-11-18

**Authors:** Felipe Lacerda de Oliveira Pessôa, Marcio Liu Sandt, Marcos Alves Correia, Carlos Rodrigo de Mello Roesler, Maria Eugenia Leite Duarte, Verônica Fernandes Vianna

**Affiliations:** 1 Orthopedics Service, Hospital Copa D'Or, Rede D'Or São Luiz, Rio de Janeiro, RJ, Brazil; 2 Biomechanical Engineering Laboratory, Hospital Universitário da Universidade Federal de Santa Catarina, Florianópolis, SC, Brazil; 3 Instituto D'Or de Pesquisa e Ensino (IDOR), Rio de Janeiro, RJ, Brazil

**Keywords:** biomechanic, humeral fractures, olecranon process, osteotomy, surgical fixation devices, biomecânica, dispositivos para fixação cirúrgica, fraturas do úmero, olécrano, osteotomia

## Abstract

**Objective:**

To compare the biomechanical characteristics of olecranon osteotomy fixation using transcortical (TC) or intramedullary (IM) screws.

**Methods:**

We sectioned synthetic polyurethane ulnas to simulate a Chevron osteotomy. Osteotomy fixations were performed using TC ( *n* = 11) or IM screws ( *n* = 11). After the fixation, we assembled the specimens on a positioning device in the testing machine to subject them to a preload of 10 N, followed by 100 loading cycles ranging from 10 to 500 N. At the end of the 100 cycles, we maintained the 500-N load and assessed the opening of the fracture focus (gap) in the osteotomy region. Then, we applied monotonic tensile loading until fixation failure and measured the maximum resistance force, system stiffness, and failure mode.

**Results:**

No group presented failure after the application of load cycles ranging from 10 to 500 N, with no difference in gap values ( *p* = 0,9420). The maximum failure force in the IM group was 1.27 times greater than in the TC group ( *p* = 0.0459). The stiffness of the 2 systems was similar ( *p* = 0,670).

**Conclusion:**

Both techniques were effective alternatives in terms of stability and rigidity. Fixation with IM screws resulted in better load-bearing capacity before failure, suggesting a potential advantage in mechanical strength. The results of the present study may help the interpretation of the clinical implications of the two techniques in investigations on fixation methods for olecranon osteotomy.

## Resumo

**Objetivo:**

Comparar as características biomecânicas da fixação da osteotomia do olécrano utilizando parafusos transcorticais (TCs) ou intramedular (IMs).

**Métodos:**

Ulnas sintéticas de poliuretano foram seccionadas simulando a osteotomia tipo Chevron. As osteotomias foram fixadas com parafusos TCs ( *n* = 11) ou IMs ( *n* = 11). Após a fixação, os corpos de prova foram montados em um dispositivo de posicionamento na máquina de ensaios e submetidos a pré-carga de 10 N, seguida por 100 ciclos de carregamento entre 10 e 500 N. Ao final dos 100 ciclos, a carga de 500 N foi mantida e a abertura do foco de fratura ( *gap* ) na região da osteotomia foi medido. A seguir, foi aplicado carregamento monotônico de tração até a falha da fixação e obtenção das medidas da força de resistência máxima, da rigidez dos sistemas e do modo de falha.

**Resultados:**

Nenhum dos grupos apresentou falha após a aplicação dos ciclos de carga entre 10 e 500 N, sem diferença nos valores do *gap* ( *p* = 0,9420). A força máxima de falha no grupo IM foi 1,27 vezes maior do que no grupo TC ( *p* = 0,0459) e a rigidez dos 2 sistemas foi semelhante ( *p* = 0,670).

**Conclusão:**

As duas técnicas mostraram ser alternativas eficazes em termos de estabilidade e rigidez. A fixação com parafuso intramedular demonstrou maior capacidade de suportar cargas antes da falha, sugerindo possível vantagem em termos de resistência mecânica. Os resultados do presente estudo poderão auxiliar na interpretação das implicações clínicas das duas técnicas nas investigações sobre métodos de fixação da osteotomia do olécrano.

## Introduction

Elbow fractures account for 7% of adult fractures, and distal humerus fractures correspond to less than half of them. 1 These fractures present a bimodal distribution pattern, affecting young men subjected to high-energy trauma and older women with bone fragility. 2 Regardless of age group, surgical treatment is the gold standard, although it is challenging due to the anatomical complexity of the elbow joint. 1 3 Conservative treatment occurs in patients with extra-articular, nondisplaced fractures, clinical contraindications to surgery, neurological deficit in the limb, and a high risk of local complications. 1

Surgical options include medial and lateral paratricipital approaches, which preserve or divide the triceps, and olecranon osteotomy, which is the preferred technique for open reduction and internal fixation of intra-articular fractures. 4 This approach provides better fracture access and superior articular visualization than nonosteotomy techniques. 4 5 6 7 8

There are several osteotomy types. 9 The most widely used is the posterior Chevron-type “V” approach, as it provides a comprehensive view of the distal humeral epiphysis and good intra-articular exposure. 1 7 8 9 10 11 12 This technique offers greater rotational stability and favors fracture consolidation and stability due to the wide contact area between bone surfaces. 4 8 However, some studies reported osteotomy-associated complications, such as pseudoarthrosis, loss of joint reduction, synthesis failure, and symptomatic implants, 12 13 14 15 motivating the search for new fixation alternatives. 4 5 6 12

Historically, tension bands have been the primary fixation technique for osteotomies due to their potential to convert triceps distraction forces into compressive forces at the fracture site. 15 However, clinical studies highlighted the advantages of osteotomy fixation with intramedullary (IM) 16 17 or transcortical (TC) screws. 7 10 Intramedullary fixation involves inserting a screw longitudinally into the olecranon medullary canal with minimal soft tissue disruption. 16 17 The lower complication rates of IM fixation may result from lower local aggression, the lack of implant migration, 16 17 18 and the lower technical complexity compared with tension bands or plates and screws. 15

Transcortical screws are an alternative for olecranon osteotomy fixation. 7 10 This technique involves inserting two TC screws perpendicularly to the osteotomy site, transfixing both cortices. 19 It is considered simpler than IM fixation and offers resistance to shearing forces, a potential advantage in the initial rehabilitation. 7

Although IM and TC fixation techniques are clinically applicable for osteotomy fixation, there is no consensus on their biomechanical performance, especially regarding fixation stability and maximum resistance force. The present study aimed to perform a biomechanical analysis in synthetic bones to compare olecranon Chevron osteotomy fixation using IM or TC screws. Evaluation parameters included resistance to cyclic loading, maximum force up to fixation failure, and assembly stiffness.

## Materials and Methods

### Composite Bones

We used 22 left ulna composite bones (Nacional Ossos, code 3020) and 2 radiopaque composite bones from the same manufacturer (code 12333) to compare the biomechanical characteristics of two osteotomy fixation systems, that is, using (i) 1 IM screw or (ii) 2 TC screws.

### Synthesis Materials and Experimental Groups

We used partially threaded cannulated screws with washers (7.0 × 90 mm) and partially threaded screws with washers (4.0 × 42 mm) from the same manufacturer (Traumédica). We randomly divided the ulnae into two groups, each undergoing a different osteotomy fixation method using one IM screw (IM group; *n* = 11) or two TC screws (TC group; *n* = 11).

### Osteotomy

Before osteotomy, markings defined the long axis of the ulna at the distal attachment site of the triceps tendon, the proximal portion of the olecranon, and the diaphyseal region. We performed the osteotomy with an oscillating saw, simulating the Chevron technique. 8 To define the area for osteotomy, we applied mold at a 140° angle, with the distal apex on the long axis (previously marked) on the ulna, 2 cm distal to the proximal bone surface of the olecranon. We did the osteotomy at a 20° angle in the sagittal plane.

### Osteotomy Fixation

#### Intramedullary Screws

**Fig. 1:**
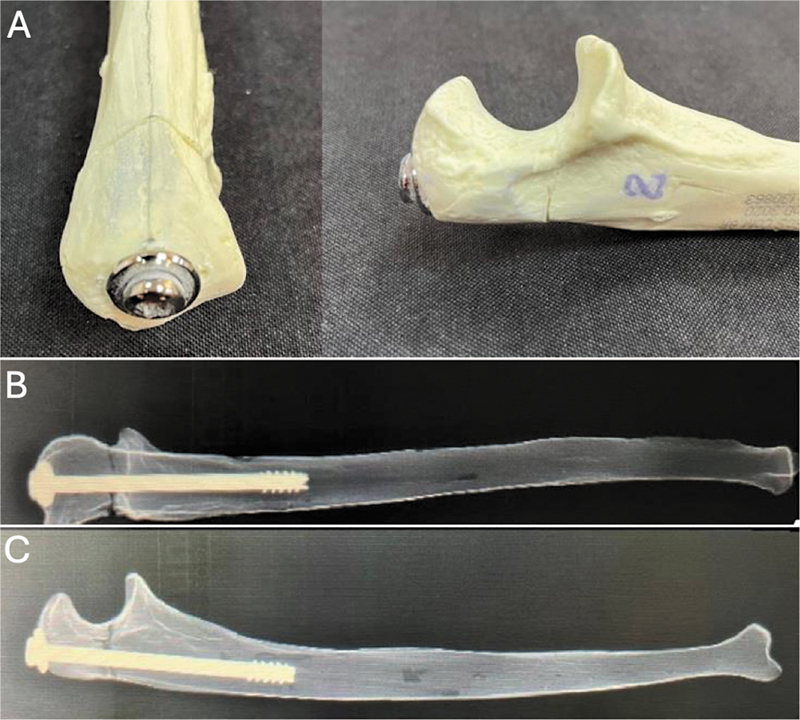
Intramedullary (IM) screw fixation. (A) Osteotomy fixation with longitudinal insertion of a cannulated screw into the medullary canal. (B) Anteroposterior and lateral (C) radiographs of radiopaque ulnae after osteotomy fixation with an IM screw.

After osteotomy reduction using a Weber bone clamp, we introduced a Kirshner wire (2.0 mm) to aid in rotational control of the fragment during assembly. The strategic position of the entry point of the guidewire into the olecranon created a path that deviated from the central axis of the bone, compensating for the physiological ulnar varus. With the guidewire in place, we drilled the entry point for the screw into the olecranon with a cannulated drill (3.2 mm). We fixated the osteotomy by inserting a cannulated screw (7.0 × 9.0 mm) with a partial thread and washer longitudinally into the medullary canal ( Fig. 1 ).

#### Transcortical Screws

**Fig. 2:**
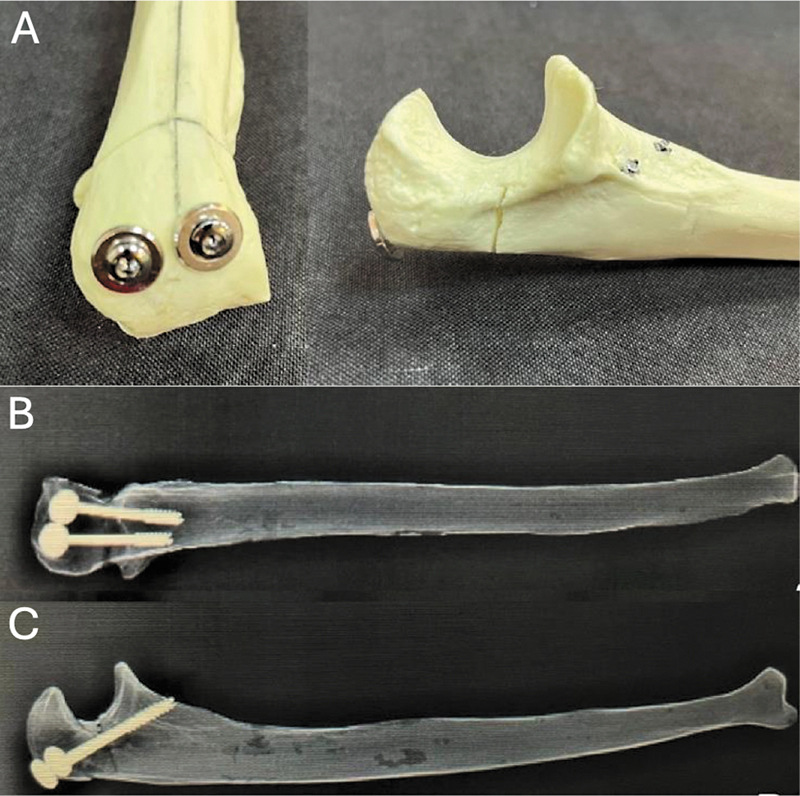
Transcortical screw (TC) fixation. (A) Osteotomy fixation by perpendicular insertion of two TC screws. (B) Anteroposterior and lateral (C) radiographs of radiopaque ulnae after osteotomy fixation with TC screws.

After reducing the osteotomy, we inserted two parallel guidewires into the proximal region of the olecranon, crossing the osteotomy area and preserving the joint region of the ulna. The distance between the wires ranged from 10 to 12 mm along the path, with the exit point located immediately anterior to the coronoid process. Through the guidewires, we made transcortical holes with a cannulated drill (2.5 mm). After reaming the holes and measuring the canal size, we fixated the osteotomy by perpendicularly inserting two screws (4 × 42–26 mm) with partial threads and washers ( Fig. 2 ).

### Biomechanical Testing

**Fig. 3:**
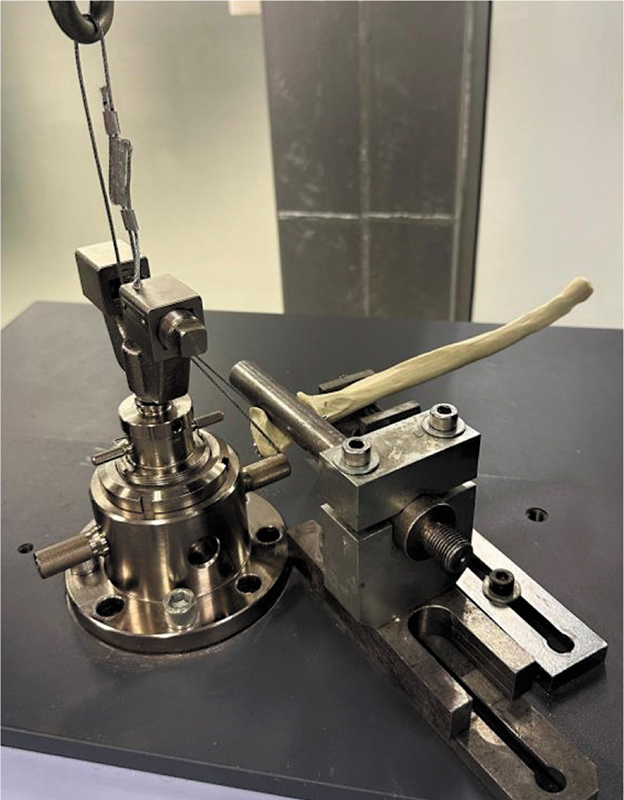
Biomechanical evaluation of fixation systems with test specimens mounted on a SHIMADZU AGS-X universal testing machine.

We evaluated the biomechanical characteristics of the fixation systems using a protocol of cyclic loading test for a predetermined number of cycles, followed by a monotonic tensile test up to the mechanical resistance limit of each assembly. We performed the experiments on a SHIMADZU AGS-X universal testing device equipped with a Shimadzu load cell and a 100 kN capacity (Shimadzu Corporation). We positioned the specimens in the testing machine using a fixation device consisting of (1) a support base fixed to the device platform, which was a reaction point for the force applied to the reconstructed fragment, and (2) a horizontal reaction rod, inserted into the joint cavity of the ulna, simulating the resistance offered by the humeral trochlea. To reproduce the action of the triceps tendon, we applied traction to the olecranon (bone fragment) using a steel cable ( Fig. 3 ).

**Fig. 4:**
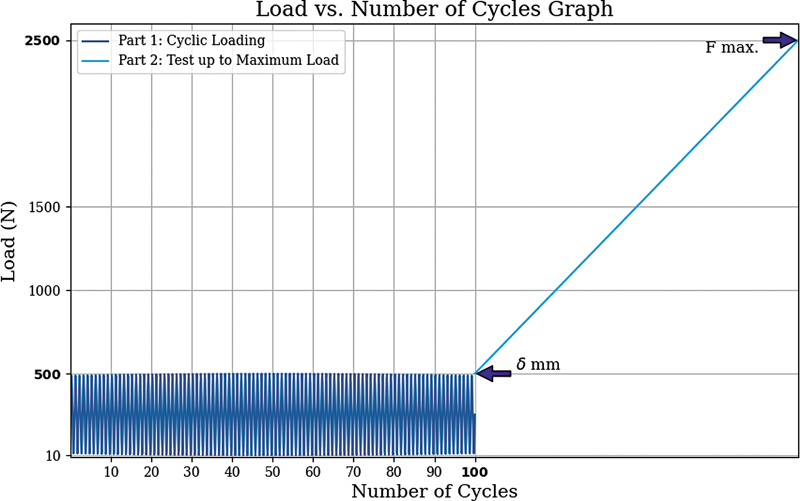
Graphical representation of the load applied to the test specimens by loading cycles.

After assembly, we applied a 10-N preload, followed by 100 loading cycles ranging from 10 to 500 N. After completing 100 cycles, we kept a 500-N load and measured the gap in the osteotomy region with a calibrating ruler. The failure criterion was osteotomy separation > 2 mm. Next, we applied a monotonic tensile loading with displacement control (10 mm/min) up to fixation failure, determining the maximum resistance force and the associated failure mode ( Fig. 4 ). We measured the stiffness of the two assemblies by calculating the slope of the force x displacement curve in the region between 520 N and 700 N.

### Statistical Analysis

A one-way analysis of variance (ANOVA) and the Student t-test compared the means of the variables from the dynamic tests and the mean failure load from the static test, assuming different variances for the osteotomy openings and equal variances for the maximum force. Statistical significance was set at *p* < 0.05. Statistical analysis was performed with RStudio, version 2024.04.2 + 764, and the R language, version 4.4.1 (Posit PBC).

## Results

### Cyclic and Quasi-Static Loading Testing

**Fig. 5:**
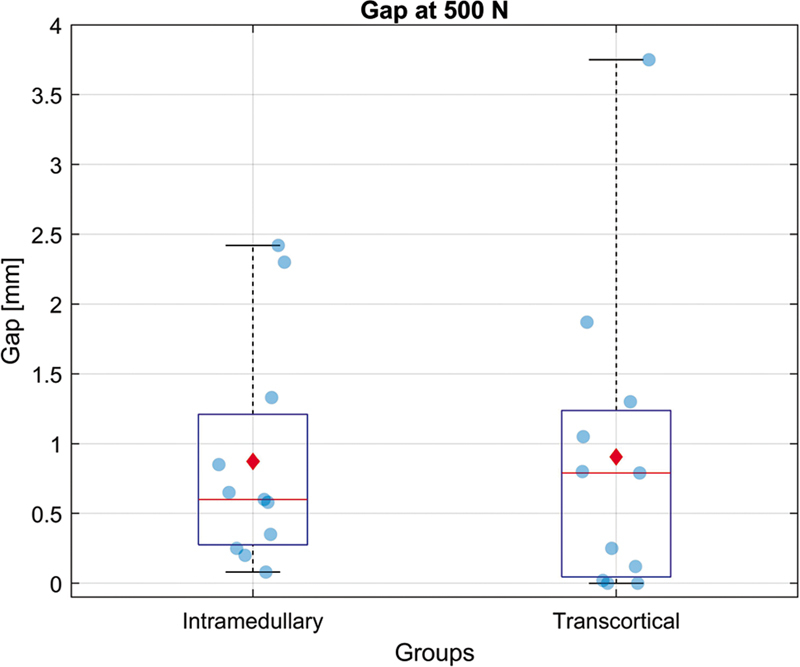
Cyclic (dynamic) loading test. Box plot showing the osteotomy opening in the two fixation methods after 100 cycles with a 500-N load. There was no failure in any assembly (gap < 2 mm). Values are expressed as median (red line), mean (red dot), and 25% and 75% percentiles (blue lines).

None of the assemblies presented failure > 2 mm after the application of the quasi-static load of 500 N, with no significant difference in gap values between the two groups ( *p* = 0.9420). Table 1 summarizes, and Fig. 5 illustrates the mean osteotomy opening values.

**Table 1:** Cyclic and quasi-static loading and maximum force test results

	Screw type		
	*Intramedullary*	*Transcortical*	*p-value* *
Osteotomy opening at 500 N (mm)	0.87 ± 0.81	0.90 ± 1.13	0.9420
Maximum force before failure (N)	1641.18 ± 304.08	1293.65 ± 442.30	0.0459
Stiffness (N/mm)	264.65 ± 38.23	258.99 ± 20.72	0.6704

**Notes** : Values expressed as mean ± standard deviation. *Student t-test.

### Maximum Resistance Test

**Fig. 6:**
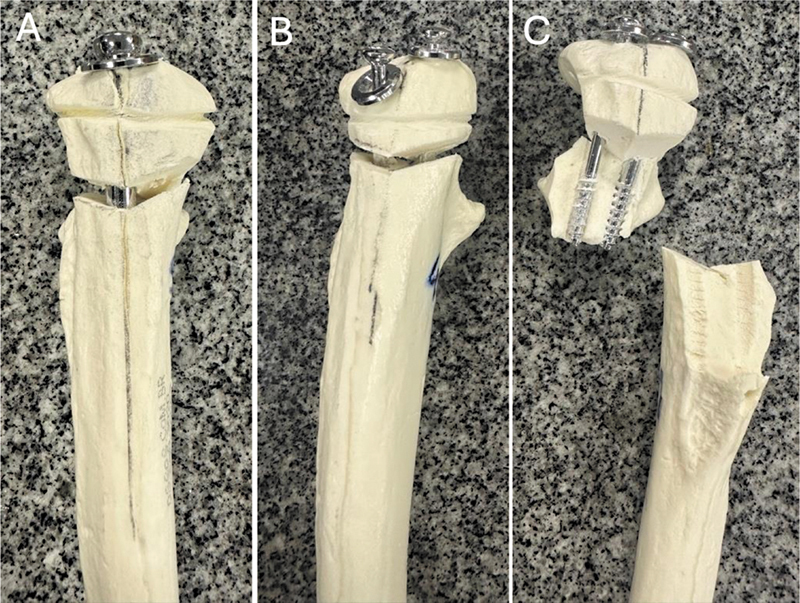
Fixation failure modes after monotonic tensile loading to determine the maximum rupture force. (A) Osteotomy opening > 2 mm in fixation with intramedullary (IM) screws or (B) transcortical (TC) screws. (C) Loosening and fracture of fragments in fixation with TC screws.

**Fig. 7:**
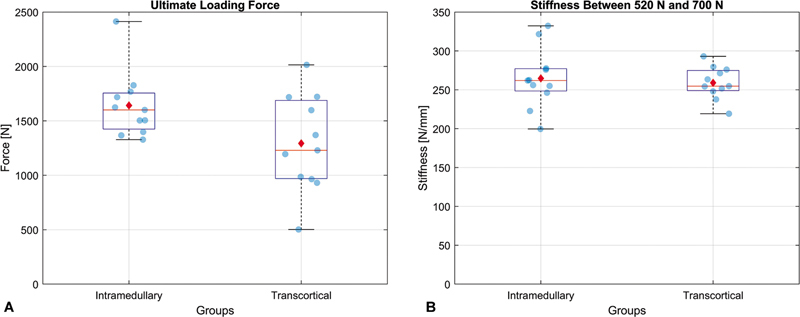
Monotonic (static) loading test. Box plot showing the maximum force before failure and the stiffness of the two osteotomy fixation methods. (A) The failure load in the intramedullary (IM) group was 1.27 times greater than in the transcortical (TC) group ( *p* = 0.0459). (B) In the region between 520 and 700 N, there was no difference between the stiffness of the two fixation systems ( *p* = 0.6704). Values are expressed as median (red line), mean (red dot), and 25% and 75% percentiles (blue lines).

After applying a monotonic tensile loading with displacement control at a speed of 10 mm/min, all specimens failed due to osteotomy displacement > 2 mm. The maximum failure force in the IM group was 1.27 times greater than in the TC group ( *p* = 0.0459). In the IM group, failures occurred mainly due to separation of the fragments in opposite directions ( Fig. 6A ). In the TC group, failures resulted from osteotomy displacement ( Fig. 6B ) and fragment fracture ( Fig. 6C ). There was no significant difference between fixation stiffness with TC or IM ( *p* = 0.67). Table 1 and Fig. 7 show the mean maximum failure forces and assembly stiffness values.

## Discussion

The present study used composite ulnas to compare the biomechanical properties of Chevron-type olecranon osteotomy fixation using one IM screw or two TC screws. After 100 tensile loading cycles ranging from 10 to 500 N, neither fixation method failed. These results suggest that both fixation methods are equally effective in supporting cyclic loads within this range of mechanical demands without compromising joint stability. In the monotonic loading test, the maximum failure force in the IM group was 1.27 times greater than in the TC group, evidencing the greater capacity of the IM system to support loads before failure and suggesting an advantage in terms of mechanical resistance.

Several clinical and biomechanical studies addressed the advantages and complications from different fixation techniques for distal humerus fractures. 4 6 12 13 17 20 Techniques involving olecranon osteotomy are widely used for open reduction and internal fixation of intra-articular fractures, as they offer better fracture visualization 4 5 Among these techniques, Chevron osteotomy is the most commonly performed as it provides greater rotational stability and an enlarged contact area between bone surfaces, optimizing local conditions for bone consolidation. 4 7 9 11 However, complications, such as nonunion, loss of reduction, and symptomatic implantation may occur. 5 12 13 14 As such, the choice of fixation techniques remain a recurring theme in the literature. 4 6 12 13

Numerous studies have investigated the stability of olecranon osteotomy fixation techniques with TC 7 10 21 22 and IM screws. 15 17 23 Wagener et al. 7 conducted one of the few biomechanical studies comparing Chevron osteotomy fixation techniques using cadaveric bones. In this study, osteotomy fixation employed TC Kirschner and tension bands or screws with or without tension bands. The biomechanical tests applied forces ranging from 200 to 500 N. The results indicated that fixation with screws alone presented rotation and translation of the proximal portion of the osteotomy under forces > 350 N. Meanwhile, the combination of screws and tension bands significantly increased the osteotomy's capacity to withstand greater forces applied to the triceps. 7 Another biomechanical study using cadaveric bones demonstrated that osteotomy fixation with compression screws and tension bands presented a better performance. 20

In the clinical context, most studies combine the outcomes of fracture fixation and osteotomy using several surgical techniques in the same case series, which challenges the critical analysis of the mechanical performance of each method. Dumartinet-Gibaud et al. 10 were the first authors to report the outcomes from the olecranon osteotomy fixation with two TC screws. In this retrospective study of 39 patients, fixation with 2 TC screws led to better clinical and radiological outcomes and lower rates of surgical revision (21%) compared with fixation using tension bands and Kirschner wires (56%). The authors also observed a lower rate of fixation loss (7% versus 24% with tension band). 10 In another retrospective study, Gill et al. 21 reported the outcomes of 27 cases, including 17 fractures and 10 olecranon osteotomies, all undergoing fixation with two TC screws. There was no loss of reduction or need for osteotomy revision, and the authors highlighted the safety, simplicity, and low complication rates of the technique. 21

An alternative to fixation with two TC screws is to use a single IM screw. A biomechanical study comparing osteotomy with IM screws or plate fixation found no significant differences in the load up to failure. 15 Cañete San Pastor et al. 17 retrospectively evaluated 26 patients with supraintercondylar fractures of the distal humerus who underwent osteotomy fixation with cannulated IM screws. After 1 year of follow-up, all osteotomies showed radiological consolidation at a mean time of 112 days, confirming the efficacy and potential superiority of the technique over other methods established in the literature. 17 Meldrum et al. 24 reported similar outcomes in a retrospective study with 92 patients, in which 37% required implant removal but none of the 10 patients with IM screw fixation required its removal. Ocalan et al. 23 also observed lower implant removal rates in osteotomies fixed with IM screws (18%) compared with plates (75%).

Our study has some limitations that are worth highlighting. In biomechanical studies, results obtained with cadaveric tissues tend to present greater translational power compared with those from artificial materials. We selected artificial ulnas made of polyurethane foam to ensure sample homogeneity, minimizing variations that could negatively influence the results. Human ulnas vary in size and degrees of osteopenia, which can challenge the accurate reproduction of results. In contrast, artificial ulnas allow greater control over the parameters analyzed in the study.

## Conclusion

Our results showed that both fixation techniques provided similar stiffness and effective cyclic stability up to 500 N, preserving the integrity of the osteotomy. Fixation with IM screws showed greater resistance to maximum load before failure, suggesting an advantage in situations of greater mechanical demand. Although the findings refer to this biomechanical model alone, they may guide clinical technical choice and future research on Chevron osteotomy.
